# Retinol-binding protein 4 in skeletal and cardiac muscle: molecular mechanisms, clinical implications, and future perspectives

**DOI:** 10.3389/fcell.2025.1587165

**Published:** 2025-04-10

**Authors:** Kangzhen Zhang, Lijuan Wang, Wei Gao, Rong Guo

**Affiliations:** ^1^ Department of Geriatrics, Nanjing Central Hospital, Nanjing, China; ^2^ Department of General Medicine, Zhongda Hospital, School of Medicine, Southeast University, Nanjing, China; ^3^ Department of Geriatrics, Zhongda Hospital, School of Medicine, Southeast University, Nanjing, China; ^4^ Department of Cardiology, Yangpu Hospital, Tongji University School of Medicine, Shanghai, China

**Keywords:** retinol-binding protein 4, skeletal muscle, cardiac muscle, insulin resistance, inflammation, cardiovascular disease, molecular mechanisms, adipokine

## Abstract

Retinol-binding protein 4 (RBP4) has emerged as a critical adipokine involved in the pathophysiology of metabolic and cardiovascular diseases. Beyond its classical role in retinol transport, RBP4 influences insulin resistance, inflammation, lipid metabolism, mitochondrial function, and cellular apoptosis in both skeletal and cardiac muscles. Elevated levels of RBP4 are associated with obesity, type 2 mellitus diabetes, and cardiovascular diseases, making it a potential biomarker and therapeutic target. This comprehensive review elucidates the molecular mechanisms by which RBP4 affects skeletal and cardiac muscle physiology. We discuss its clinical implications as a biomarker for disease risk and progression, explore therapeutic strategies targeting RBP4, and highlight future research directions. Understanding the multifaceted roles of RBP4 could pave the way for novel interventions against metabolic and cardiovascular disorders.

## 1 Introduction

Retinol-binding protein 4 (RBP4) is a 21-kDa secreted protein belonging to the lipocalin family, primarily synthesized in the liver and adipose tissue, serving as the main transport protein for retinol (vitamin A alcohol) in the bloodstream (Kanai et al., 1968; Peterson, 1971; Blaner, 1989). Beyond its traditional role, RBP4 functions as an adipokine involved in regulating glucose metabolism and insulin sensitivity (Yang et al., 2005; Graham et al., 2006). Elevated RBP4 levels have been observed in insulin-resistant states such as obesity and type 2 diabetes mellitus (T2DM), implicating it in the development of metabolic syndrome (Klöting et al., 2007; Kovacs et al., 2007; Olsen and Blomhoff, 2020; Flores-Cortez et al., 2022). Skeletal muscle, accounting for approximately 80% of insulin-stimulated glucose disposal, is critical for glucose uptake and utilization (DeFronzo and Tripathy, 2009). Cardiac muscle relies on efficient energy metabolism for continuous contractile activity. Dysregulation of RBP4 affects both muscle types, leading to insulin resistance in skeletal muscle and contributing to cardiac remodeling, hypertrophy, and dysfunction (Nono and Blüher, 2021; Li et al., 2024; Ji et al., 2022; Schiborn et al., 2022). Recent studies highlight RBP4’s role in promoting cardiomyocyte injury and pyroptosis post-myocardial infarction and its association with adverse cardiovascular events (Zhang et al., 2021; Zhou et al., 2021; Qian et al., 2022; Chen et al., 2021). Understanding RBP4’s mechanisms in these tissues is crucial for developing targeted therapies for metabolic and cardiovascular diseases. The study of RBP4 has evolved significantly since its initial characterization, incorporating insights from various disciplines, including endocrinology, immunology, and molecular biology. This interdisciplinary approach has been crucial in unraveling the complex roles of RBP4 in metabolic and cardiovascular health. As we delve into the molecular mechanisms and clinical implications of RBP4, it is important to consider both the advancements and the challenges in this field of research.

## 2 Molecular mechanisms of RBP4 in skeletal muscle

RBP4 affects skeletal muscle through a complex network of molecular interactions, influencing insulin signaling, inflammation, lipid metabolism, and muscle physiology. These mechanisms are intricately intertwined, creating a multifaceted impact on muscle function and metabolic health. The following sections will explore each of these aspects in detail, highlighting the latest research findings and their implications for muscle biology.

### 2.1 Impairment of insulin signaling

RBP4 impairs insulin signaling by interfering with insulin receptor substrate (IRS) proteins. Elevated RBP4 levels reduce tyrosine phosphorylation of IRS-1, diminishing its ability to activate downstream signaling molecules like phosphatidylinositol 3-kinase (PI3K) and protein kinase B (Akt) (Craig et al., 2007; Janke et al., 2006). This results in decreased glucose transporter type 4 (GLUT4) translocation to the plasma membrane, reducing glucose uptake (Suh et al., 2010). RBP4 may induce suppression of cytokine signaling 3, which binds to IRS-1 and inhibits its activation (Norseen et al., 2012). Additionally, RBP4 activates c-Jun N-terminal kinase (JNK), leading to serine phosphorylation of IRS-1, further impairing insulin signaling (Sabio and Davis, 2010). Downregulation of GLUT4 expression involves inhibition of myocyte enhancer factor 2 and GLUT4 enhancer factor, key transcription factors regulating GLUT4 gene expression (Zorzano et al., 2005; Handschin and Spiegelman, 2008). Moreover, RBP4 affects insulin signaling by activating the retinol transport receptor STRA6, leading to downstream effects on JAK2/STAT3 signaling pathways (Huang et al., 2021; Perumalsamy et al., 2021), promoting gene expression that interferes with insulin action.

### 2.2 Promotion of inflammation

Chronic low-grade inflammation is a hallmark of insulin resistance. RBP4 stimulates pro-inflammatory cytokines such as tumor necrosis factor-alpha (TNF-α), interleukin-6 (IL-6), and interleukin-1β (IL-1β) in skeletal muscle cells and infiltrating macrophages (Moraes-Vieira et al., 2014; Hotamisligil, 2006). These cytokines activate inhibitory kinases such as IκB kinase (IKK) and JNK, which phosphorylate IRS-1 on serine residues, impairing insulin signaling (Nono and Blüher, 2021). RBP4 binds to toll-like receptor 4 on muscle cells, triggering nuclear factor kappa-light-chain-enhancer of activated B cells (NF-κB) and mitogen-activated protein kinases (MAPKs) pathways, promoting inflammatory gene transcription and contributing to insulin resistance (Flores-Cortez et al., 2022). This suggests RBP4 acts as a pro-inflammatory mediator, linking metabolic stress to inflammatory pathways. Numerous clinical studies have demonstrated strong correlations between elevated RBP4 levels and increased inflammatory markers, including high-sensitivity C-reactive protein (hsCRP), TNF-α, and IL-6 (Bujak and Frangogiannis, 2007a). Importantly, longitudinal interventional studies have demonstrated that reductions in RBP4 levels through exercise, dietary modifications, or combined interventions are accompanied by parallel improvements in metabolic parameters, suggesting a causal relationship rather than a mere association (Flores-Cortez et al., 2022; Ghorbanian et al., 2023a; Ghorbanian and Saberi, 2021). For instance, Ghorbanian et al. (2022) demonstrated that a 12-week aerobic exercise intervention significantly reduced serum RBP4 levels by approximately 11% (P = 0.001) in men with metabolic syndrome, while a combined intervention with ketogenic diet produced an even more pronounced reduction of 23.1% (P = 0.020). These reductions in RBP4 levels were significantly correlated with improvements in insulin resistance markers, as evidenced by decreased HOMA-IR values (P = 0.001). Notably, when comparing intervention groups, both ketogenic diet alone (P = 0.041) and combined exercise-diet intervention (P = 0.017) resulted in significantly lower RBP4 levels compared to controls, highlighting the potential therapeutic value of targeting RBP4 through lifestyle interventions (Ghorbanian et al., 2023a). RBP4 also activates antigen-presenting cells, leading to adipose tissue inflammation and systemic insulin resistance (Schiborn et al., 2022), and the muscle-adipose tissue crosstalk mediated by RBP4 exacerbates metabolic dysfunction.

### 2.3 Alteration of lipid metabolism

Altered lipid metabolism contributes to insulin resistance. RBP4 suppresses peroxisome proliferator-activated receptor alpha (PPARα) and its target genes involved in fatty acid β-oxidation, like carnitine palmitoyltransferase I (Amengual et al., 2008; Carrasco et al., 2023). This leads to the accumulation of intramyocellular lipids and intermediates like diacylglycerol and ceramides, activating protein kinase C isoforms that phosphorylate IRS-1 on serine residues, inhibiting insulin signaling (Itani et al., 2002). RBP4 upregulates sterol regulatory element-binding protein 1c, enhancing lipogenic enzyme expression and contributing to lipid accumulation and insulin resistance (Eberlé et al., 2004). Additionally, RBP4 may interfere with adipose triglyceride lipase and hormone-sensitive lipase, which are crucial for lipid mobilization, disrupting lipid homeostasis (Steinhoff et al., 2024) and exacerbating ectopic lipid deposition in skeletal muscle.

### 2.4 Impact on mitochondrial function and oxidative stress

RBP4 decreases peroxisome proliferator-activated receptor gamma coactivator 1-alpha (PGC-1α), a regulator of mitochondrial biogenesis (Patti et al., 2003), leading to reduced mitochondrial content, decreased oxidative capacity, and energy production. Mitochondrial dysfunction results in the overproduction of reactive oxygen species (ROS) (Anderson et al., 2009), causing oxidative damage to cellular components and activating stress kinases such as JNK and IKK, further impairing insulin signaling (Houstis et al., 2006). RBP4 may impair mitochondrial dynamics by affecting fusion and fission balance, leading to fragmented mitochondria and reduced adenosine triphosphate (ATP) production (Jheng et al., 2012), exacerbating metabolic dysfunction in skeletal muscle.

### 2.5 Influence on muscle fiber type and composition

RBP4 influences muscle fiber type distribution by promoting a shift from oxidative type I fibers to glycolytic type II fibers (Schiaffino and Reggiani, 2011). Glycolytic fibers are less efficient in glucose oxidation and more susceptible to insulin resistance. RBP4 may downregulate PGC-1α and affect transcription factors involved in fiber type specification (Lin et al., 2005), reducing oxidative capacity and impairing endurance, which may affect muscle performance and contribute to fatigue in metabolic disorders. The shift in muscle fiber type composition induced by RBP4 not only affects metabolic properties but also has significant implications for muscle strength and endurance. Type I fibers, which are more oxidative, are crucial for sustained, low-intensity activities, while type II fibers are essential for high-intensity, short-duration activities. The RBP4-induced shift toward type II fibers may contribute to reduced endurance capacity and increased fatigability, potentially exacerbating the physical limitations often observed in metabolic disorders (Schiaffino and Reggiani, 2011; Lin et al., 2005). This interplay between RBP4 and muscle fiber type adds another layer to our understanding of how metabolic dysregulation can impact overall physical function and quality of life.

### 2.6 Modulation of autophagy

RBP4 impairs autophagy by activating the mechanistic target of rapamycin complex 1, inhibiting autophagy initiation (Mizushima and Komatsu, 2011). Impaired autophagy leads to the accumulation of dysfunctional mitochondria and proteins, contributing to insulin resistance, oxidative stress, and inflammation, further impairing metabolic function. Enhancing autophagy may represent a therapeutic strategy to mitigate RBP4-induced metabolic dysfunction.

### 2.7 Induction of muscle atrophy and pyroptosis

RBP4 promotes muscle-specific ubiquitin ligases, such as atrogin-1 and muscle RING-finger protein-1, increasing protein degradation (Bodine et al., 2001). Zhang et al. demonstrated that RBP4 exacerbates denervation-induced muscle atrophy via the STRA6-dependent pathway (Zhang et al., 2024). RBP4 induces pyroptosis in muscle cells by interacting with NOD-, LRR- and pyrin domain-containing protein 3 (NLRP3) inflammasomes (Zhang et al., 2021; Zhang et al., 2024), contributing to muscle cell loss and atrophy. Pyroptosis amplifies inflammatory responses, leading to further tissue damage and functional impairment.

**FIGURE 1:**
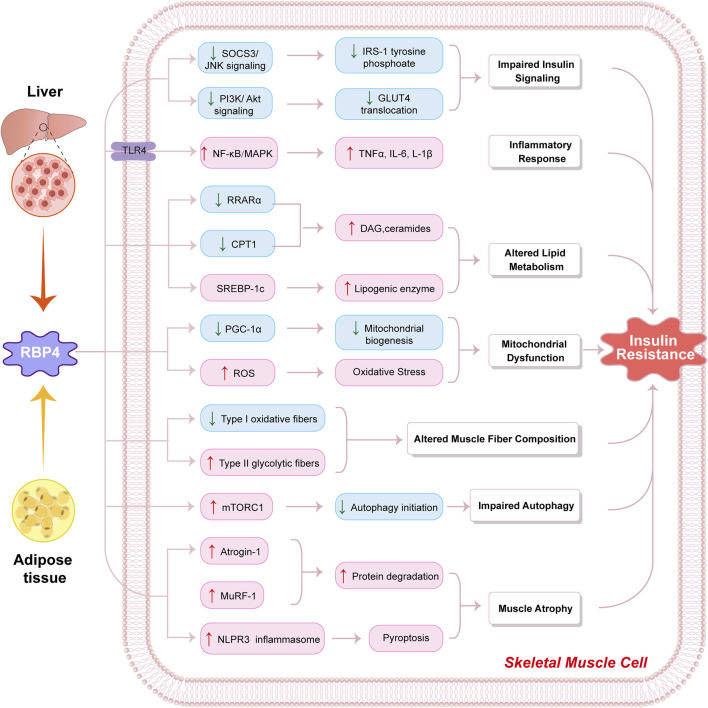
Molecular mechanisms by which RBP4 influences skeletal muscle metabolism. Schematic illustration of how retinol-binding protein 4 (RBP4) derived from liver and adipose tissue induces insulin resistance in skeletal muscle cells through multiple pathways. RBP4 triggers: (1) Impaired insulin signaling via decreased SOCS3/JNK and PI3K/Akt signaling, leading to reduced IRS-1 tyrosine phosphorylation and GLUT4 translocation; (2) Inflammatory response through TLR4-mediated activation of NF-κB/MAPK pathway, resulting in increased production of inflammatory cytokines (TNFα, IL-6, L-1β); (3) Altered lipid metabolism through downregulation of RRARα and CPT1, and activation of SREBP-1c, leading to increased DAG/ceramides and lipogenic enzyme levels; (4) Mitochondrial dysfunction via decreased PGC-1α-mediated mitochondrial biogenesis and increased ROS production causing oxidative stress; (5) Altered muscle fiber composition showing decreased Type I oxidative fibers and increased Type II glycolytic fibers; (6) Impaired autophagy through mTORC1 activation and reduced autophagy initiation; and (7) Muscle atrophy via upregulation of Atrogin-1, MuRF-1, and NLPR3 inflammasome activation leading to increased protein degradation and pyroptosis. These molecular alterations collectively contribute to the development of insulin resistance in skeletal muscle cells. Blue boxes indicate decreased expression/activity; pink boxes indicate increased expression/activity. Arrows indicate activation/induction; lines indicate inhibition.

To clearly illustrate the multiple pathways through which RBP4 affects skeletal muscle, Figure 1 provides a schematic representation of the molecular mechanisms by which RBP4 mediates insulin resistance in skeletal muscle cells.

## 3 Molecular mechanisms of RBP4 in cardiac muscle

In cardiac muscle, RBP4 plays a crucial role in various pathophysiological processes, including cardiac remodeling, fibrosis, oxidative stress, and energy metabolism. The effects of RBP4 on the heart are multifaceted, involving complex signaling cascades and interactions with various cellular components. Understanding these mechanisms is essential for developing targeted therapies for cardiovascular diseases associated with metabolic dysfunction.

### 3.1 Promotion of cardiac hypertrophy

RBP4 promotes cardiomyocyte hypertrophy through activation of the MAPK/ERK pathway, stimulating ERK1/2 phosphorylation and enhancing hypertrophic gene transcription (Liu et al., 2021; Gao et al., 2016), increasing expression of atrial natriuretic factor and brain natriuretic peptide. RBP4 stimulates the PI3K/Akt/mTOR pathway, leading to increased protein synthesis and cell growth (Molkentin, 2004). RBP4 may increase intracellular calcium levels, activating calcineurin, which dephosphorylates the nuclear factor of activated T-cells, promoting hypertrophic gene expression (Wilkins and Molkentin, 2004). Additionally, RBP4 can modulate microRNA expression in cardiomyocytes, influencing gene networks associated with hypertrophy and fibrosis (Zhao et al., 2021), adding an epigenetic layer to its impact on cardiac remodeling.

#### 3.1.1 Induction of cardiac fibrosis

RBP4 increases transforming growth factor-beta one and connective tissue growth factor expression in cardiac fibroblasts (Bujak and Frangogiannis, 2007b), stimulating fibroblast activation and collagen synthesis. RBP4 alters matrix metalloproteinase activity, disrupting the balance between matrix degradation and synthesis (Lindsey et al., 2016), and may influence the phenotypic transformation of vascular smooth muscle cells under high-glucose conditions via the RhoA/ROCK1 pathway (Zhou et al., 2023), contributing to vascular remodeling and stiffness. The resulting fibrosis impairs myocardial compliance and contributes to diastolic dysfunction.

### 3.2 Enhancement of oxidative stress and apoptosis

RBP4 enhances ROS production by activating NADPH oxidase components (Takac et al., 2011) and impairing electron transport chain function (Madamanchi and Runge, 2007), leading to excess ROS. Elevated ROS levels induce oxidative damage and activate apoptotic pathways in cardiomyocytes. RBP4 modulates Bcl-2 family proteins, decreasing anti-apoptotic Bcl-2 and increasing pro-apoptotic Bax, leading to caspase activation (Whelan et al., 2012; Elmore, 2007). The intrinsic apoptotic pathway culminates in cardiomyocyte death, contributing to cardiac dysfunction and remodeling.

### 3.3 Impairment of endothelial function

RBP4 reduces endothelial nitric oxide synthase expression and activity, decreasing nitric oxide (NO) production (Kim et al., 2006), leading to vasoconstriction and impaired vasodilation. RBP4 increases adhesion molecules and chemokine expression on endothelial cells (Gimbrone and García-Cardeña, 2016), facilitating leukocyte adhesion and contributing to atherogenesis. Elevated RBP4 levels are associated with increased arterial stiffness (Armeni et al., 2021), underscoring its role in vascular aging and stiffness.

#### 3.3.1 Disruption of calcium homeostasis

RBP4 affects sarcoplasmic reticulum Ca^2+^-ATPase, decreasing its expression and impairing calcium reuptake (Bers, 2002), leading to diastolic calcium overload and systolic dysfunction. It alters the ryanodine receptor function, affecting calcium release during excitation-contraction coupling (Hegyi et al., 2019). Disrupted calcium homeostasis activates pathways promoting hypertrophic gene expression (Wilkins and Molkentin, 2004; Sag et al., 2009), contributing to cardiac dysfunction.

##### 3.3.1.1 Alteration of cardiac energy metabolism

RBP4 decreases PPARα activity, reducing fatty acid oxidation gene expression (Finck and Kelly, 2006) and shifting substrate utilization toward glucose metabolism. Under stress conditions, reliance on glucose metabolism is less efficient and may exacerbate energy deficits. RBP4 impairs mitochondrial biogenesis and function, reducing ATP production and contributing to energy deficits in the myocardium (Lesnefsky et al., 2001), impairing contractile function, and promoting pathological remodeling.

#### 3.3.2 Induction of cardiomyocyte pyroptosis

RBP4 induces pyroptosis in cardiomyocytes by interacting with the NLRP3 inflammasome, leading to gasdermin D cleavage (Zhang et al., 2021; Wang et al., 2019), resulting in inflammatory cell death and releasing pro-inflammatory cytokines such as IL-1β and IL-18. This contributes to cardiac injury post-myocardial infarction, amplifies inflammation, exacerbates tissue damage, and impairs cardiac repair mechanisms. The diverse effects of RBP4 on cardiac muscle highlight the complex interplay between metabolic dysfunction and cardiovascular health. The ability of RBP4 to induce both hypertrophy and cell death (through apoptosis and pyroptosis) underscores the delicate balance in cardiac physiology and the potential for RBP4 to tip this balance toward pathological outcomes. Moreover, the impact of RBP4 on cardiac energy metabolism suggests a potential link between systemic metabolic disorders and heart failure, a connection that warrants further investigation (Finck and Kelly, 2006; Lesnefsky et al., 2001; Wang et al., 2019).

**FIGURE 2:**
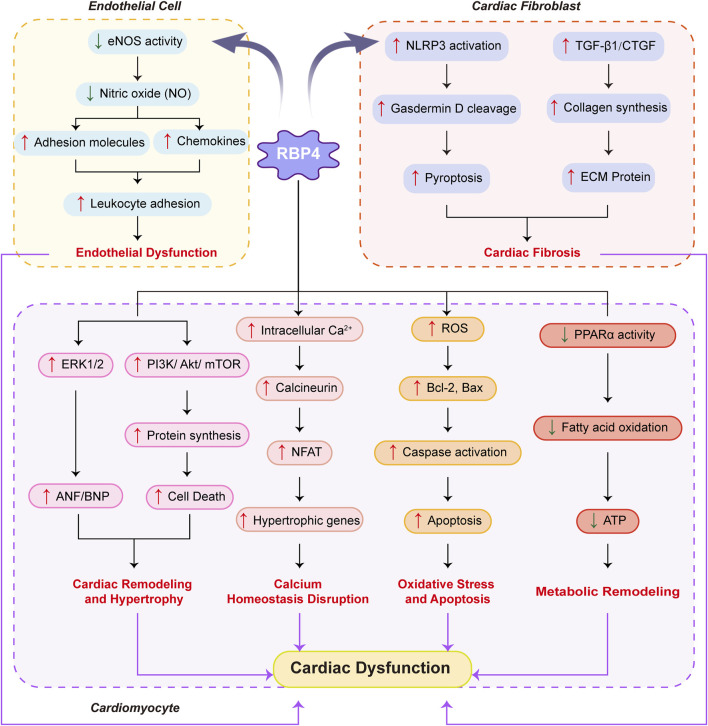
Molecular mechanisms by which RBP4 affects cardiac muscle.Schematic representation of how RBP4 mediates cardiac dysfunction through its effects on endothelial cells, cardiac fibroblasts, and cardiomyocytes. In endothelial cells, RBP4 decreases eNOS activity and NO production, leading to increased adhesion molecules and chemokines, resulting in enhanced leukocyte adhesion and endothelial dysfunction. In cardiac fibroblasts, RBP4 activates NLRP3 inflammasome and increases TGF-β1/CTGF expression, promoting gasdermin D cleavage, pyroptosis, collagen synthesis, and ECM protein production, ultimately causing cardiac fibrosis. In cardiomyocytes, RBP4 triggers four major pathological pathways: (1) Cardiac remodeling and hypertrophy through activation of ERK1/2 and PI3K/Akt/mTOR signaling, leading to increased ANF/BNP expression and protein synthesis; (2) Calcium homeostasis disruption via increased intracellular Ca2+ and calcineurin/NFAT signaling, inducing hypertrophic gene expression; (3) Oxidative stress and apoptosis through elevated ROS production, Bcl-2/Bax dysregulation, and caspase activation; and (4) Metabolic remodeling via decreased PPARα activity, resulting in reduced fatty acid oxidation and ATP production. These pathological changes collectively contribute to cardiac dysfunction. Blue arrows indicate decreased expression/activity; red arrows indicate increased expression/activity. Solid arrows represent direct effects; dashed lines indicate regulatory relationships.

Figure 2 comprehensively depicts the molecular mechanisms through which RBP4 induces cardiac dysfunction via its multifaceted effects on endothelial cells, cardiac fibroblasts, and cardiomyocytes.

## 4 Clinical implications

Elevated serum RBP4 levels are associated with insulin resistance and T2DM, correlating with impaired glucose tolerance and hyperinsulinemia (Takebayashi et al., 2007; Cho et al., 2006). Meta-analyses confirm the association between high RBP4 levels and increased T2DM risk (Tan et al., 2024; Zabetian-Targhi et al., 2015). In obesity and metabolic syndrome, RBP4 levels correlate with markers of metabolic dysregulation (Nono and Blüher, 2021; Dadej et al., 2022). Beyond statistical associations, multiple lines of evidence establish RBP4 as an independent prognostic risk factor for cardiovascular disease. Prospective cohort studies with multivariate adjustment for traditional risk factors demonstrate that elevated RBP4 levels independently predict future adverse cardiovascular events with hazard ratios ranging from 1.80 to 3.26 for intermediate and high RBP4-based scores, respectively (Qian et al., 2022; Chen et al., 2021). Ye et al. (2023) developed and validated an RBP4-based multimarker score that significantly improved risk prediction accuracy beyond established risk factors (net reclassification improvement of 0.24, 95% CI, 0.15–0.34; P < 0.001). Mechanistically, the direct pathophysiological effects of RBP4 on vascular inflammation, endothelial dysfunction, and cardiac remodeling provide strong biological plausibility for its causal role in cardiovascular pathogenesis, as evidenced by interventional studies showing that modulation of RBP4 levels directly affects cardiovascular outcomes (Ji et al., 2022; Schiborn et al., 2022; Li et al., 2020). High RBP4 levels predict coronary artery disease (Qian et al., 2022; Perumalsamy et al., 2021), correlate with atherosclerosis severity (Chen et al., 2021; Ye et al., 2023), and are associated with cardiac remodeling in heart failure (Li et al., 2020; Christou et al., 2023). RBP4 levels are increased in gestational diabetes mellitus (Wu et al., 2022; Leca et al., 2024), non-alcoholic fatty liver disease (NAFLD) (Huang and Xu, 2022; Hu et al., 2023), and may be linked to neurodegenerative diseases such as amyotrophic lateral sclerosis (Yosry et al., 2022). Additionally, RBP4 levels are associated with carotid intima-media thickness, suggesting a role in early atherosclerosis (Rychter et al., 2024), and serve as a potential biomarker for sarcopenia in older adults, correlating with changes in lean mass (Chang et al., 2023).

Pharmacological interventions targeting RBP4 include fenretinide, a synthetic retinoid reducing RBP4 synthesis and secretion, thereby improving insulin sensitivity (Napoli, 2012); thiazolidinediones decreasing RBP4 levels by activating PPARγ (Cho et al., 2006); and DPP-4 inhibitors that may reduce RBP4 levels and exert anti-inflammatory effects (Kim et al., 2015). The development of RBP4 antagonists, such as small molecules or antibodies inhibiting RBP4 function, shows promise in preclinical studies (Kim and Priefer, 2021; Oluwamodupe and Adeleye, 2023). Targeting the RBP4-STRA6 signaling pathway offers therapeutic potential in mitigating insulin resistance and β-cell dysfunction (Huang et al., 2021; Perumalsamy et al., 2021). Lifestyle modifications, including dietary interventions and exercise, reduce RBP4 levels and improve metabolic parameters (Dadej et al., 2022; Ghorbanian et al., 2023b). High-intensity interval training decreases RBP4 levels and improves insulin sensitivity in metabolic syndrome (Ghorbanian and Saberi, 2023a), and weight loss through bariatric surgery also reduces RBP4 levels and improves metabolic outcomes (Hany et al., 2022).

Measuring RBP4 levels aids in identifying individuals at risk for metabolic and cardiovascular diseases, with changes in RBP4 levels reflecting intervention effectiveness. Combining RBP4 with other biomarkers improves prognostic accuracy for adverse cardiovascular events (Flores-Cortez et al., 2022; Ye et al., 2023), and an RBP4-based multimarker score has been proposed as a prognostic tool for patients with acute coronary syndrome (Ye et al., 2023). RBP4 could be integrated into multimarker panels for risk stratification and monitoring disease progression. While the potential of RBP4 as a biomarker is promising, it is important to note the challenges in its clinical application. The lack of standardized measurement methods and established reference ranges across different populations presents a significant hurdle (Takebayashi et al., 2007; Cho et al., 2006). Additionally, the specificity of RBP4 as a marker for particular diseases or conditions remains a subject of debate, given its involvement in multiple physiological and pathological processes (Tan et al., 2024; Zabetian-Targhi et al., 2015). Future research should focus on developing and validating robust, standardized assays for RBP4 quantification and establishing population-specific reference values to enhance its clinical utility.

## 5 Conclusion

RBP4 significantly impacts skeletal and cardiac muscle physiology through diverse molecular mechanisms. Its involvement in insulin resistance, inflammation, lipid metabolism, mitochondrial dysfunction, and apoptosis underscores its importance in metabolic and cardiovascular diseases. RBP4 serves as a valuable biomarker and a promising therapeutic target. Integrating findings from extensive research enhances our understanding and highlights potential interventions. Future research focusing on unraveling its complex interactions and developing targeted therapies holds great promise for improving patient outcomes. As our understanding of RBP4 continues to evolve, this protein sits at the intersection of multiple physiological and pathological processes. The complexity of RBP4’s actions underscores the need for a systems biology approach to fully elucidate its roles and potential as a therapeutic target. While significant progress has been made, many questions remain unanswered, particularly regarding the long-term effects of RBP4 modulation and its interactions with other metabolic regulators. Future research will undoubtedly continue to unravel the intricate web of RBP4’s actions, potentially revolutionizing our approach to metabolic and cardiovascular diseases.

## 6 Future directions

Future research should focus on three key areas while maintaining comprehensive investigation. First, elucidating molecular mechanisms through identifying specific receptors beyond STRA6 (Huang et al., 2021; Zhang et al., 2024), and characterizing downstream signaling pathways. Advanced technologies like CRISPR-Cas9 will be valuable for understanding tissue-specific RBP4 functions and potential off-target effects of therapies (Rychter et al., 2024; Yang et al., 2021). Second, Developing RBP4-targeted therapeutics including small-molecule antagonists and antibodies (Kim and Priefer, 2021; Oluwamodupe and Adeleye, 2023), while exploring synergistic effects with existing treatments. The RBP4-STRA6 signaling pathway offers particular promise (Huang et al., 2021; Perumalsamy et al., 2021). Third, integrating RBP4 assessment into personalized medicine by identifying disease-associated RBP4 gene polymorphisms (Rychter et al., 2024; Yang et al., 2021). and incorporating RBP4 into risk prediction models for patient stratification. Additional exploration of RBP4’s role in neurodegenerative disorders (Yosry et al., 2022), immune regulation (Yang et al., 2021), and its interplay with other adipokines in epicardial adipose tissue (Christou et al., 2023) may reveal novel intervention targets, as could investigation of its effects on vascular smooth muscle cell phenotypic transformation (Zhou et al., 2023).
